# Mechanisms of Ion Channels Voltage-Dependency: All about Molecular Sensors, Gates, Levers, Locks, and Grease

**DOI:** 10.3389/fphar.2012.00174

**Published:** 2012-09-27

**Authors:** Gildas Loussouarn, Mounir Tarek

**Affiliations:** ^1^Institut National de la Santé et de la Recherche Médicale, UMR1087Nantes, France; ^2^Centre National de la Recherche Scientifique, UMR 6291Nantes, France; ^3^L’institut du Thorax, L’UNAM Université, Université de NantesNantes, France; ^4^Equipe de Chimie et Biochimie Théoriques, UMR Synthèse et Réactivité de Systèmes Moléculaires Complexes, Centre National de la Recherche Scientifique, Université de LorraineNancy, France

Given the wealth of electrophysiological, biochemical, optical, and structural data regarding ion channels voltage-dependency, we decided to put together in this special issue, up to date reviews describing the molecular details of these complex voltage-gated channels (and in one instance voltage-dependent phosphatases: Villalba-Galea, 2012). The articles focus mostly on the molecular mechanisms underlying channels voltage-dependency, such as the electromechanical coupling governing their activation, but also on molecular mechanisms governing their regulation by lipids. We anticipate that such knowledge will help one to better understand the pathophysiology of channelopathies (Choveau et al., 2012; Delemotte et al., 2012; Jurkat-Rott et al., 2012) and lead to new pharmacological approaches.

Molecular mechanisms underlying voltage-dependent activation and inactivation are complex, especially because channels are behaving in drastically different ways. Many reviews included in the present Research Topic issue describe models that rationalize these different behaviors:

– In some channels, e.g., HCN, KAT, activation is promoted by hyperpolarization while in others, e.g., Kv channels, it is promoted by depolarization, despite a similar global structure and behavior of their voltage sensors. The opposite behavior may come from different kinds of S4-S5/S6 interactions, that can be transient for hyperpolarization activated channel, permanent for depolarization activated channel (Blunck and Batulan, 2012), or bimodal, with the residues implicated in the S4-S5/S6 interaction being different in the open and closed states (Choveau et al., 2012). Along the same lines, the peculiar closed state inactivation observed in Kv4 channels may also come from a transient S4-S5/S6 interaction (Bähring et al., 2012).
– Forced uncoupling between the voltage sensor and the pore leads to opposite effects: this uncoupling favors channel closure of Shaker channels or, conversely, opening of the Kv-KcsA chimeric and KCNQ1 channels. This is most probably due to intrinsic properties of the pore, favoring a closed state in the former case and an open state in the latter (Blunck and Batulan, 2012; Vardanyan and Pongs, 2012).
– The nature of the gating motion of S6 falls into two categories as described in details by Labro and Snyders (2012). This may due to different constraints associated with the origin of the main stimulus, which comes from either the nearby voltage sensor domain or from a distal part of the C-terminus. C-terminal domains of Kv channels are indeed critical for the modulation of channel gating by signal transduction elements (Barros et al., 2012). These two categories may also be related with the intrinsic properties of the pore mentioned above (Vardanyan and Pongs, 2012).
– hERG is a very peculiar channel with slow activation gate and fast inactivation gate. Several molecular mechanisms (differences in voltage sensor dynamics, in the strength of S4-S5/S6 coupling, modulatory role of the N- and C-termini) may be at the origin of that peculiar behavior (Cheng and Claydon, 2012).

Finally, in addition to the pore forming subunits, membrane lipids (Choveau et al., 2012; Moreno et al., 2012; Rodríguez Menchaca et al., 2012), intracellular ions (Goodchild and Fedida, 2012), and β-subunits (Sun et al., 2012) that can associate with multiple stoichiometry (Wrobel et al., 2012) also modulate the channel voltage-dependency.

We hope that this series of reviews will bring researcher in the field (electrophysiologists, biochemists, modelers), a compendium of the knowledge gathered so far on the complex mechanisms of ion channel/enzyme voltage-dependency.
